# 73061 The UAB COVID-19 Collaborative Outcomes Research Enterprise (CORE): Developing a Learning Health System in Response to the Global Pandemic

**DOI:** 10.1017/cts.2021.728

**Published:** 2021-03-30

**Authors:** Jami L. Anderson, Becky Reamey, Emily Levitan, Alia Tunagur, Michael J. Mugavero

**Affiliations:** University of Alabama at Birmingham

## Abstract

ABSTRACT IMPACT: Interdisciplinary networks represent critical components of translational science and learning system development. Our work impacts translational research by presenting an evidence-based approach to developing interdisciplinary networks in response to the COVID-19 pandemic; the approach presented may have broad applications within other academic institutions and medical centers. OBJECTIVES/GOALS: As a local response to the COVID-19 pandemic, we established the University of Alabama at Birmingham COVID-19 Collaborative Outcomes Research Enterprise (CORE) as an interdisciplinary learning health system (LHS) to achieve an integrated health services and outcomes research response amid the pandemic. METHODS/STUDY POPULATION: We adapted a learning system framework, based upon a scoping review of the literature and the Knowledge to Action Framework for implementation science. Leveraging this framework, we developed an institutional-level collaborative network of extant expertise and resources to rapidly develop an interdisciplinary response to COVID-19. The network was designed to quickly collect newly published or clinical information related to COVID-19, to evaluate potential usefulness of this information, and to disseminate the new knowledge throughout the interdisciplinary network; we strove to engage a wide variety of expertise and skills in the network. Thus, we subsequently used social network analysis to examine the emergence of informal work patterns and diversified network capabilities based on the LHS framework. RESULTS/ANTICIPATED RESULTS: We identified three principal characteristics of institutional LHS development including: 1.) identifying network components; 2.) building the institutional collaborative network; and 3.) diversifying network capabilities. Seven critical components of LHS were identified including: 1.) collaborative and executive leadership, 2.) research coordinating committee, 3.) oversight and ethics committee, 4.) thematic scientific working groups, 5.) programmatic working groups, 6.) informatics capabilities, and 7.) patient advisory groups. Evolving from the topical interests of the initial CORE participants, three scientific working groups (health disparities, neurocognition, and critical care) were developed to support the learning network. DISCUSSION/SIGNIFICANCE OF FINDINGS: Interdisciplinary collaborative networks are critical to the development of LHS. The COVID-19 CORE LHS framework served as a foundational resource that may support further institutional-level efforts to develop responsive learning networks. The LHS approach presented may have broad applications within other academic institutions and centers.

